# Factors in the Mental Health of Children from Low-Income Families in the United States: An Application of the Multiple Disadvantage Model

**DOI:** 10.3390/ejihpe15120253

**Published:** 2025-12-11

**Authors:** Tyrone C. Cheng, Celia C. Lo

**Affiliations:** 1 School of Social Work, University of Alabama, Little Hall, Tuscaloosa, AL 35401, USA; 2 Peraton, Defense Personnel and Security Research Center, Seaside, CA 93955, USA; celiaclo@yahoo.com

**Keywords:** low-income children, mental health, parenting, peers, access to care

## Abstract

**Objective:** This study on children in low-income families explored whether their mental health problems are attributable to distress from five socioeconomic disadvantage factors playing roles in the multiple disadvantage model. These factors are social disorganization, social structural factors, social relationships, health/mental health, and access to care factors. **Methods:** The present study employed data extracted from the 2021 National Survey of Children’s Health, describing 7540 low-income children. Weighted logistic regression was conducted (with robust standard errors). **Results:** It showed that such children were more likely to have mental health problems when seven variables were present. The variables were argumentative children, parents’ difficulty with parenting, children’s difficult peer relations, children being bullied, families’ problematic substance use, families’ use of public health insurance, and families’ difficulty accessing mental health services. In turn, children were less likely to have mental health problems in the presence of six variables: a rundown neighborhood, an unsafe neighborhood, children’s Hispanic ethnicity, children’s Asian ethnicity, children’s general good health, and parents’ good mental health. The present study’s findings support the multiple disadvantage model. **Conclusions:** That is, the five types of factors key to the model (social disorganization, social structural, social relationships, health/mental health, and access to care) were observed to be related to low-income children’s mental health problems. These findings’ three main implications for practice are that it is crucial to (*a*) ensure children receive mental health services they need; (*b*) facilitate effective parent–child communication; and (*c*) provide low-income families with psychoeducation. Their main implications for policy involve two domains. Improving physical environments and safety in poor neighborhoods is necessary, as is enforcing schools’ anti-bullying rules and using schools to foster students’ assertiveness.

## 1. Introduction

Of children in the United States’ general population during 2021–2022, mental health problems like depression, anxiety, and behavior disorders characterized 15.9–23.3%, according to the literature reports (Centers for Disease Control and Prevention, 2025; Substance Abuse and Mental Health Services Administration, 2023). However, among the low-income children in that group, specifically, a wider range of rates was reported, 13.2–40.7% (Drabick et al., 2021; Howell, 2004; Keeton et al., 2023; Lynch & Clemans-Cope, 2023; Tyrell et al., 2024). The higher rates of mental health problems suggested by this wider range (which adds higher percentages in more numbers than it adds lower ones) probably relate to the low-income children’s wider experience of socioeconomic disadvantages.

**Figure 1:**
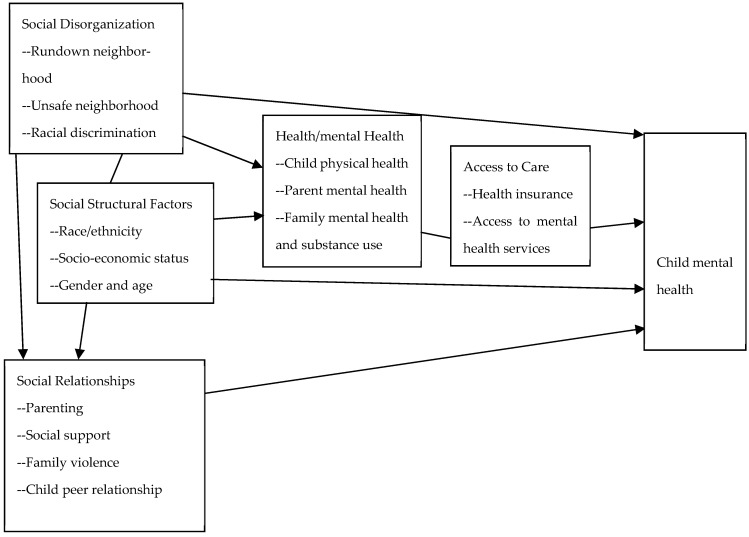
The multiple disadvantage model explaining low-income children’s mental health.

The present study proposed that the five types of socioeconomic disadvantages central to the *multiple disadvantage model* create distress in low-income children and foster mental health problems among them. The model was initially developed to investigate racial disparities in violent victimization (Lo et al., 2013, 2015) with five types of socioeconomic disadvantage factors (social disorganization, social structural, social relationships, health/mental health, access to care; see Figure 1). The multiple disadvantage model has been applied to explain children’s mental health (Cheng & Lo, 2025a), adolescents’ behavioral problems (Cheng & Li, 2017; Cheng & Lo, 2017; Li & Cheng, 2017), access to mental health services (Cheng & Lo, 2025b), and access to substance use treatment (Kedia et al., 2021).

### 1.1. Literature Review

#### 1.1.1. Social Disorganization

Social disorganization is a socioeconomic disadvantage, sometimes manifesting as residency in a rundown or unsafe neighborhood. Prior studies with small samples (*n* = 95 to *n* = 491) reported that social disorganization could generate distress that contributes to the mental health problems of low-income children (Buckner et al., 2004; Odgers & Russell, 2017; Tyrell et al., 2024). Social disorganization can, at times, also manifest as historical or structural racism, which has an undesirable impact on individuals of minority ethnicity in the general population (Lo et al., 2013). Experiencing racial discrimination may frustrate low-income children and adversely affect their mental health. While prior studies of social structural factors as they pertain to low-income children’s mental health were not numerous, their results seem to support the speculation that residing in a rundown and/or unsafe neighborhood, as well as facing racial discrimination, negatively affects mental health in low-income children.

#### 1.1.2. Social Structural Factors

The social structural factor of race/ethnicity also has a possible role in the mental health of low-income children. One prior study in Philadelphia found that low-income African American children were more likely than low-income White children to exhibit a mental health problem (Hillemeier et al., 2007). Another study with a small sample (*n* = 95) of low-income children found Hispanic children to be less likely than White children to have a mental health problem (Buckner et al., 2004), while still another small study (*n* = 344) of low-income children reported no significant difference in mental health between Hispanic and White children (Hill et al., 2003). Other extant research with, yet again, a small sample (*n* = 95) observed no association between low-income children’s mental health and the demographic characteristics of gender and age (Buckner et al., 2004). Even these mixed prior results were sufficient to lead the present researchers to propose that those low-income children most likely to have mental health problems would also have a minority ethnicity.

Low-income parents’ education, employment status, and income are further social structural factors related to their children’s mental health. One prior study in an urban area in the northeastern U.S. found that low-income children were less likely to have mental health problems if their parents had relatively more education (Tyrell et al., 2024). Another study sampling the population in Boston, Chicago, and San Antonio found low-income children of employed parents to actually be less likely to have mental health problems (despite reporting that the adults’ work schedules could prove disadvantageous) (Chase-Lansdale et al., 2003). A different study, from San Francisco, did not observe such an association between low-income children’s mental health and their parents’ employment (Keeton et al., 2023). Published research on low-income samples also shows that children in families with relatively more financial resources have less risk of developing a mental health problem. The studies derived this result whether employing a small sample (*n* = 34 to *n* = 491) (Dufford et al., 2019; Tyrell et al., 2024) or, in one case, a national sample (Y. C. Wang & McLeroy, 2023). In contrast, one published study with a small sample (*n* = 344) reported no such association describing low-income children (Hill et al., 2003). In sum, the published research suggests that low-income children’s risk of mental health problems is likely greater if their parents are unemployed and lack much education or income.

#### 1.1.3. Social Relationships

For low-income families living with multiple socioeconomic disadvantages, the associated distress may be mitigated by supportive social relationships. According to prior studies with small samples (*n* = 344 to *n* = 749) (Hill et al., 2003; White et al., 2012), children of low-income parents having supportive social networks that contributed to effective parenting were less likely to have mental health problems than children of parents without such networks. Additionally, one study with a small sample (*n* = 491) reported that low-income children’s mental health problems were associated in a positive direction with single-parent families (Tyrell et al., 2024). This suggests that spousal support in parenting is significant to the parents’ ability to nurture their children’s mental health. Moreover, earlier research on small samples (*n* = 95 to *n* = 344) reported that family conflict and domestic violence increase low-income children’s risk of mental health problems (Buckner et al., 2004; Hill et al., 2003). At least one small study (*n* = 104) found that low-income children whose peer relationships were difficult were relatively likely to have a mental health problem (Drabick et al., 2021), and numerous studies of the general population indicated that children who had been bullied were indeed likely to have such a problem (Aarons et al., 2001; Cheng & Lo, 2022a; Goldbach et al., 2018; Kowalski & Limber, 2013; Sterzing et al., 2020). Prior analyses of social relationship factors tend to support the argument that low-income children’s mental health is associated in a negative direction with spousal support, family support, and social support.

#### 1.1.4. Health/Mental Health

The literature suggests that low-income children’s mental health is often connected to their physical health as well as to their parents’ mental health problems, attributable, in part, to the socioeconomic disadvantages parents face. Prior studies with the general population (Acosta-Pérez et al., 2012; Cheng & Lo, 2025a; Isasi et al., 2016; McKelvey et al., 2011; Olazagasti et al., 2012) indicated that children’s physical health was associated with their risk for mental health problems. Additionally, parents who had mental health problems were not likely to parent supportively (Waylen & Stewart-Brown, 2010) in the manner essential to children’s good mental health. Indeed, several studies with small samples (*n* = 95 to *n* = 432) (Buckner et al., 2004; Keeton et al., 2023; Zacher et al., 2022) and one study with a national sample (Y. C. Wang & McLeroy, 2023) found an association in a positive direction between low-income parents’ mental health problems and their children’s mental health problems. Moreover, earlier research in the general population found that just one in ten depressive mothers sought treatment for depression (Cheng & Lo, 2016; Woolhouse et al., 2009). The challenges of experiencing multiple socioeconomic disadvantages may foster both depression and alcohol or drug misuse among low-income parents. Either disorder can render parents unlikely to parent effectively, which may elevate children’s risk of mental health problems. All of these findings seem to support the present researchers’ estimation that low-income children’s poor health, as well as their parents’ poor health or poor mental health, can diminish the children’s mental health.

#### 1.1.5. Access to Care

First, it appears that the literature on access to care factors as they relate to mental health problems in low-income children is rather limited. Among the socioeconomic disadvantages that low-income families may face is a lack of medical insurance. However, one prior study of a small sample of low-income children (*n* = 432) found no association between insured status and mental health (Keeton et al., 2023). Accessing needed mental health services was nevertheless difficult for low-income families (Bringewatt & Gershoff, 2010; Copeland & Snyder, 2011; Drabick et al., 2021; Santiago et al., 2013). Low-income children’s access could, for instance, be deterred by the unavailability of services nearby, or by a lack of transportation to available services, or by a required out-of-pocket payment for services (Cheng & Lo, 2025b; Koroloff et al., 1994, 1996; Overhage et al., 2024). Once again, however, there was published research with a small sample of low-income children with a mental health problem (*n* = 432) that reported they found no link between that problem and a family’s difficulty with getting the required services (Keeton et al., 2023). The multiple disadvantage model holds that a lack of medical insurance and a lack of access to needed mental health services are disadvantages that create distress and foster mental health problems in children. For that reason, the present researchers posit that, among the sampled low-income children, mental health will be associated in a positive direction with their being insured and with their receipt of mental health services.

### 1.2. Hypotheses

The bulk of the relevant literature available comprises studies whose small samples of low-income children limit the generalizability of the findings. Within that literature, findings on, specifically, social structural factors’ roles in children’s mental health are mixed. There is, moreover, a scarcity of data quantifying these children’s experience of racial discrimination and of bullying victimization; data describing low-income children’s general health and their parents’ substance use are also scarce.

The present study aimed to address these deficiencies, proposing two hypotheses. The first was that low-income children’s mental health problems would be associated in a positive direction with social disorganization (e.g., neighborhoods’ adverse physical conditions, unsafe neighborhoods, racial discrimination); with social structural factors (e.g., minority race/ethnicity); with social relationships (e.g., single mothers, family conflict, and bullying victimization); with parents’ mental health problems and substance use; and with a lack of medical insurance. The second was that low-income children’s mental health problems would be associated in a negative direction with other social structural factors (e.g., parents’ education, parents’ employment, and family income); with other social relationships (e.g., spousal support, family support, and social support); with children’s physical health; and with children’s access to mental health services.

## 2. Methods

### 2.1. Sample

The present secondary analysis extracted a nationally representative sample of 7540 low-income children in the United States from the public-use 2021 National Survey of Children’s Health (NSCH) dataset, which sampled 50,892 children in all. With mailed and emailed questionnaires and phone interviews, NSCH researchers collected information on health and mental health status, behaviors, family relationships, social relationships, medical insurance coverage, access to health and mental health services, and the neighborhood of residency (U.S. Census Bureau, 2022) from caregivers. The present sample exclusively included children aged 6–17 years, who resided with their biological parents or stepparents and whose family income fell below 200% of the federal poverty level. For missing data quantifying continuous variables, the present researchers substituted it with the average measure for that variable across the entire sample. For missing data for dichotomous (yes/no) variables, the present researchers substituted it with a “no” response. Because the present study applied the public-use NSCH data, it was exempt from the requirement to obtain institutional review board approval. The authors have no competing interests to declare that are relevant to the content of this study.

### 2.2. Measures

The present study’s outcome variable was the dichotomous *child mental health problem* (yes/no). It indicated whether, at the time of the survey, the child reportedly had been diagnosed with depression, anxiety, ADHD, behavioral/conduct problems, autism spectrum disorder, and/or Tourette’s syndrome. This study also employed explanatory variables that are classified into five groups: social disorganization, social structural factors, social relationships, health and mental health, and access to care.

The first group of explanatory variables, describing social disorganization, included a *rundown neighborhood* (yes/no), indicated by parent perception of the neighborhood of the child’s residency as “litter or garbage on street or sidewalk,” “vandalism such as broken windows or graffiti,” and/or “poorly kept or rundown housing.” Also, the first group included an *unsafe neighborhood*, whose measure involved a 4-point scale that parents employed to agree (or not) that the neighborhood of the child’s residency was unsafe for children. Anchors of the scale were 1 (*definitely disagree*), 2 (*somewhat disagree*), 3 (*somewhat agree*), and 4 (*definitely agree*). The first group of explanatory factors also included *racial discrimination* (yes/no), indicating whether children had ever been judged or treated unfairly due to their race/ethnicity.

The second group of explanatory variables characterized children’s and parents’ social structural factors. This group included the dummy variables *Black*, *Hispanic*, *Asian*, *other racial/ethnic minority*, and *non-Hispanic White* (the reference), indicating children’s race/ethnicity. As well, *parent education* was described using nine levels: 1 (*8th grade or below*), 2 (*9th–12th grade*), 3 (*graduated high school or GED*), 4 (*vocational school*), 5 (*some college*), 6 (*associate degree*), 7 (*undergraduate degree*), 8 (*master’s degree*), and 9 (*doctoral or professional degree*). Additionally, *employed parent* (yes/no) described parents employed during 50 of the 52 weeks preceding an NSCH interview. Also, part of the second group of explanatory variables, *family income-to-poverty ratio* (provided by NSCH), represented the percentage of the federal poverty level (FPL) that a family’s income was estimated at. The second group also included some demographic characteristics, serving as control variables. They were *girl* (versus *boy*), a *child aged 6–10 years*, a *child aged 11–13 years*, a *child aged 14–17 years* (the reference), and a *parent’s age* (in years).

Social relationship measures made up the third group of explanatory variables. *Single mother* (yes/no) represented households that were headed by an unmarried female parent. *Family violence* (yes/no) reported if a child had ever witnessed the parent being punched, kicked, hit, or slapped by a partner in the child’s home. *Neighbor support* was measured via the total score from three survey items that employed a 4-point response scale (Cronbach’s α = 0.79). The three items reflected a parent’s perception that other adults in the neighborhood (*a*) provide help to others, (*b*) watch out for each other’s children, and (*c*) know where to get help. Anchors of the scale were 1 (*definitely disagree*), 2 (*somewhat disagree*), 3 (*somewhat agree*), and 4 (*definitely agree*). A higher total score indicated a stronger supportive network of neighbors. *Poor parent–child communication* assessed how well the parent and child shared ideas or talked about important things, again using a 4-point response scale. The scale’s anchors were 1 (*very well*), 2 (*somewhat well*), 3 (*not very well*), and 4 (*not well at all*). The variable *argumentative child* represented parents’ accounting of how often their children argued with them; another 4-point response scale was employed: 1 (*never*), 2 (*sometimes*), 3 (*usually*), and 4 (*always*).

The third group of explanatory variables also included *the difficulty of parenting a child*. This measure, with a 5-point response scale, constituted parents’ estimates of how often it was difficult for them to function as the child’s parent: 1 (*never*), 2 (*rarely*), 3 (*sometimes*), 4 (*usually*), and 5 (*always*). Parents used the scale to respond to three survey items (Cronbach’s α = 0.80). The three were “… really bothers me,” “… hard to care for,” and “… angry with the child.” A higher total score indicated greater difficulty experienced in parenting a child. *Child’s difficulty with peers* employed a 3-point scale with which parents gauged children’s difficulty in making and keeping friends: 1 (*no difficulty*), 2 (*a little difficulty*), and 3 (*a lot of difficulty*). Finally, *being bullied* (yes/no) indicated whether a child had reportedly been bullied, picked on, or excluded by peers at least once in the 12 months preceding the interview.

A fourth group of explanatory variables in the present study characterized children’s health and parents’ mental health. *Child’s general health* was assessed with five responses: 1 (*poor*), 2 (*fair*), 3 (*good*), 4 (*very good*), and 5 (*excellent*). *Parent’s mental health* was self-reported via a 5-point scale: 1 (*poor*), 2 (*fair*), 3 (*good*), 4 (*very good*), and 5 (*excellent*). *Family mental health problem* (yes/no) indicated whether a child had ever lived with someone who was mentally ill. *Family substance use problem* (yes/no) denoted if a child had ever lived with someone who had an alcohol-use problem and/or drug-use problem.

The fifth group of explanatory variables described children’s access to care. First, families’ medical insurance coverage was described using four dichotomous variables: *uninsured* (the reference), *public health insurance*, *private health insurance*, and *other health insurance*. *Public* insurance meant Medicaid, Medical Assistance, or some other public medical insurance program, while *private* insurance meant that medical insurance was either purchased independently by parents or sponsored by parents’ employers. In addition, the variable *difficulty accessing mental health services* (yes/no) denoted whether parents found it difficult to gain access to any mental health services their children needed.

### 2.3. Data Analysis

In light of its binary outcome variable, the present study applied STATA logistic regression featuring linearized variance estimations with robust standard errors, along with sampling weights provided in the NSCH dataset. All explanatory variables were entered simultaneously into the logistic regression modeling. Preliminary analyses of the VIF (2.09 or lower) and correlations (−0.45 ≤ *r* ≤ 0.49) confirmed no multicollinearity problems characterizing the explanatory variables.

## 3. Results

### 3.1. Descriptive Statistics

Of the 7540 low-income children, over one-third (36.7%) had mental health problems, nearly one-third (31.4%) resided in rundown neighborhoods, and less than one-tenth (6.0%) experienced racial discrimination (see Table 1). On average, parents or caregivers reportedly “definitely disagree[d]” (score of 1.5) that their neighborhoods were unsafe for children. Low-income children in the sample included more than half (51.4%) who were non-Hispanic Whites, as well as Blacks (11.6%), Hispanics (22.5%), Asians (5.8%), and those of other racial/ethnic minority backgrounds (8.7%). Less than one-third of the sampled parents (32.1%) were single mothers. The sampled parents’ average education was at the vocational school level (or 4.9); the average family income was 112.2% of the federal poverty level, with a majority (65.1%) of parents reportedly employed. In addition, 47.1% of the sampled children were girls. As well, the largest proportion (38.7%) of the children were 6–10 years old, the smallest proportion (24.7%) were 11–13 years old, and those ages 14–17 constituted 36.6%. The average age of parents was 41.7 years.

On average, neighbor support scored 9.4 (of 12.0 possible), and difficulty parenting child scored 5.3 (of 15.0 possible). Moreover, parent–child communication averaged *very well* (or 1.5) and, on average, children were *sometimes* argumentative (or 2.1). Additionally, 8.0% of the sampled children had witnessed the specified violence between household members in the home. On average, children had *no difficulty* with peers (or 1.3). At the same time, over one-third (34.5%) of the sampled children reportedly had been bullied. Children’s health was *very good* on average (scoring 4.4); parents’ average mental health was *good* (or 3.8). Of the low-income children’s family members, 13.4% reportedly had at least one mental health problem, and 13.0% reportedly had a substance use problem. In addition, over half of the children (56.5%) were covered by public health insurance, over one-third (37.9%) were covered by private health insurance, 4.7% were covered by other health insurance, and 7.7% were uninsured. Difficulty accessing needed mental health services had reportedly been experienced by less than one-tenth (9.8%) of these low-income families.

### 3.2. Multivariate Analysis

The results of the multivariate analysis confirmed the significant difference between the hypothesized model and the null model (Wald’s χ^2^ = 562.49, *p* < 0.01; see Table 2). Children were less likely to have mental health problems when their home was in a rundown neighborhood (OR = 0.73; *p* < 0.05) or an unsafe neighborhood (OR = 0.78; *p* < 0.05). Their likelihood of having a mental health problem showed no association, however, with experiencing racial discrimination. Concerning the sampled children’s race/ethnicity, the present study observed that Asian children (OR = 0.31; *p* < 0.01) and Hispanic children (OR = 0.72; *p* < 0.05) were less likely than non-Hispanic White children to have a mental health problem. In addition, girls (OR = 0.58, *p* < 0.01) were less likely to have mental health problems than boys. No other social structural factor or demographic characteristic in this study exhibited a significant association with the outcome, children’s mental health problems.

Several measures of social relationships, in turn, were associated with a relatively higher likelihood of the mental health problem outcome. The measures were having an argumentative child (OR = 1.17, *p* < 0.05); having relative difficulty in parenting the child (OR = 1.13, *p* < 0.01); reporting the child’s relative difficulty with peers (OR = 3.29, *p* < 0.01); and the child being bullied (OR = 1.61, *p* < 0.01). Furthermore, for the sampled low-income children, children’s general health (OR = 0.74, *p* < 0.01) and parents’ mental health (OR = 0.84, *p* < 0.01) were associated negatively with children’s risk of a mental health problem. Additionally, a family substance use problem (OR = 1.58, *p* < 0.01) was associated in a positive direction with such a risk. Coverage by a public health insurance program (OR = 1.78, *p* < 0.01) and difficulty accessing needed mental health services (OR = 7.70, *p* < 0.01) elevated the likelihood of these low-income children’s mental health problems by 78% and 670%, respectively. Other types of medical insurance were not significantly associated with the outcome variable in this study.

## 4. Discussion

The analysis of the 2021 NSCH data indicated 36.7% of the sampled low-income children reportedly had mental health problems. This percentage exceeded some others (e.g., 13.2–25.0%), observed by prior research with low-income children (Drabick et al., 2021; Howell, 2004; Lynch & Clemans-Cope, 2023; Tyrell et al., 2024). Among children reported to have mental health problems, 9.9% had depression, 17.1% had anxiety, 16.2% had ADHD, 14.0% had a behavioral/conduct problem, and 20.5% had some other mental health problem.

The present results supported the first hypothesis in that low-income children’s mental health problems proved to be associated in a positive direction with their having been bullied, with parents’ mental health problems, with family members’ substance use, and with a lack of any insurance. The results also supported the second hypothesis in that low-income children’s mental health problems proved to be associated in a negative direction with children’s health and with access to mental health services. However, the results also showed that other associations hypothesized by this study either lay in the opposite direction or were not statistically significant.

### 4.1. Social Disorganization

The results showed that residency in a rundown or unsafe neighborhood was linked to a reduced risk of reported mental health problems, challenging some prior results obtained with small samples (*n* = 95 to *n* = 151) of homeless families (Buckner et al., 2004) and of children with behavioral problems (Odgers & Russell, 2017). A plausible explanation of this present unexpected finding is that low-income children may be resilient as they face the distress that their neighborhoods’ poor physical conditions and unsafe environments create.

### 4.2. Social Structural Factors

Despite the unexpected findings on the negative association between rundown/unsafe neighborhoods and children’s mental health problems mentioned earlier, a closer examination of the present data showed an interaction term involving a rundown neighborhood, unsafe neighborhood, and Black child (OR = 1.56, *p* < 0.01) to yield an association in a positive direction with the odds that children had a mental health problem. This further calculation echoed a prior study’s finding for the majority-Black child population in a larger city in the northeastern U.S. (Tyrell et al., 2024). A plausible explanation of the interaction term result is that, compared to children who are not Black, low-income Black children experience worse mental/emotional distress arising from residency in rundown and/or unsafe neighborhoods.

On the other hand, this study found no impact on the outcome variable made by the sampled children’s experience of racial discrimination. It did determine that 16.7% of White child respondents, 27.7% of Hispanic child respondents, 25.5% of Black child respondents, 7.0% of Asian child respondents, and 23.1% of child respondents reporting other racial/ethnic background reported experiencing such discrimination. Furthermore, a close examination of the present data indicated that an interaction term between Black child and rundown neighborhood, unsafe neighborhood, and racial discrimination (OR = 1.90, *p* < 0.05) was associated in a positive direction with the risk of children’s mental health problems. Moreover, an identical examination of an interaction term between sampled Hispanic children and rundown neighborhood, unsafe neighborhood, and racial discrimination (OR = 1.77, *p* < 0.05) revealed a significant association with the outcome, also in a positive direction. Such findings suggest that Black children and Hispanic children who live in disadvantaged, dangerous neighborhoods may be relatively vulnerable to certain adverse effects on mental health arising out of racially discriminatory experiences.

To recap, low-income Black children and Hispanic children living in rundown or unsafe neighborhoods were found to be vulnerable to negative mental health impacts. To counter these impacts, communities and cities should strive to improve neighborhoods’ physical environments and to add new safety measures. Launching or strengthening community policing programs and neighborhood watch groups can prove effective (De Marco & Vernon-Feagans, 2013; Dukes et al., 2009). As well, educational institutions should foster racial harmony and bullying awareness throughout their communities to help minimize low-income students’ experiences of racial discrimination and bullying (Crouch et al., 2023; Goldweber et al., 2013; Hass, 2022; Jenson et al., 2013). Schools should enforce anti-bully rules and promote assertiveness, bystander support, and the use of “stop signals” among students (Masho et al., 2019; Ross & Horner, 2014).

The results of testing the present social structural factors showed that Asian children were 68.8% less likely to have been diagnosed with a mental health problem when compared to non-Hispanic White children. This marked difference contradicted the findings about race/ethnicity and low-income children that several prior studies of regional (Hillemeier et al., 2007) or otherwise small samples (*n* = 95 to *n* = 344) (Buckner et al., 2004; Hill et al., 2003) reported. One plausible explanation is Asian ethnic communities’ openness to relatively informal sources of help when a mental health issue arises. For instance, Asian children with mental health problems often consult herbalists or folk healers for help (Nguyen et al., 2011; Spencer et al., 2010). Moreover, Asian cultures often stigmatize mental health problems. Some Asian respondents have been hesitant even to acknowledge children’s mental health symptoms; Asian parents and children alike may be reluctant to consult mental health professionals for a diagnosis (Abe-Kim et al., 2007; Cheng & Lo, 2022b; C. Wang et al., 2019; Zhang et al., 2020).

Similarly, the present results indicated that Hispanic children were 28.3% less likely than non-Hispanic White children to have a mental health diagnosis. Such a finding was consistent with prior results (Buckner et al., 2004). A plausible explanation is that Hispanic parents lack much confidence in the medical establishment, and they tend to use self-diagnosis, lay healers, and herbal remedies (Coffman et al., 2008).

Looking more deeply into the present data, the researchers derived an interaction term between racial discrimination and children aged 11–13 years (OR = 0.44, *p* < 0.05) that demonstrated an association in a negative direction with children’s likelihood of mental health problems. This means that, in the present study, children aged 14–17 years old were more likely than younger children to experience racial discrimination and find it mentally distressing. Older children, of course, tend to join more often than younger ones in various social activities, which exposes them to a greater risk of experiencing racial discrimination. It is plausible that this makes them more prone to mental health problems than younger children.

### 4.3. Social Relationships

The results on social relationship factors here confirmed that higher measures for the variables, *difficulty parenting child* and *argumentative child*, were associated with increases in these children’s risk for mental health problems; these results supported some prior findings (Hill et al., 2003; White et al., 2012). In contrast, the present analysis found no significant association between the risk of mental health problems and the variable *neighbor support*. Challenging the results of some earlier research with small samples (*n* = 95 to *n* = 49) (Buckner et al., 2004; Hill et al., 2003; Tyrell et al., 2024), the present study demonstrated that low-income children’s mental health problems were not associated with the factors of *single mother* and *family violence*. These findings on social relationships suggest that effective parenting and nurturing parent–child relationships are the keys to children’s mental health.

### 4.4. Health/Mental Health

The findings showed that low-income children’s risk of mental health problems, however, increased 229% among those who had difficulty relating to thier peers; as well, the risk rose to 61% among children experiencing bullying victimization. Both these findings were consistent with prior results sampling the general population (Aarons et al., 2001; Cheng & Lo, 2022a; Drabick et al., 2021; Goldbach et al., 2018; Kowalski & Limber, 2013; Sterzing et al., 2020). Overall, these findings suggest that low-income children’s mental health can suffer when children are argumentative with parents and have peer relationships marked by conflict. Family intervention, then, is most likely to succeed when it fosters effective communication between children and parents and includes psychoeducation that builds parents’ knowledge of and coping skills addressing children’s mental health problems (Hoagwood et al., 2010).

In addition, the findings on low-income children’s health and parents’ health/mental health demonstrated associations in a negative direction between children’s risk of mental health problems and their general health, and also between their risk and parents’ mental health. The finding involving parents was consistent with prior research (Buckner et al., 2004; Keeton et al., 2023; Y. C. Wang & McLeroy, 2023; Zacher et al., 2022). An association in a positive direction, however, was observed here between the outcome variable and family members’ substance use; the suggestion is that the factors crucial to low-income children’s good mental health include parents’ mental health, household members’ substance use, and children’s physical health. Social workers and therapists should, then, be alert to parents’ potential mental health problems and families’ potential substance use. Where it is indicated, professionals should refer family members for professional treatment.

### 4.5. Access to Care

The results for the access to care factors included a determination that difficulty accessing mental health services was the factor that was most strongly associated with the outcome variable in this study. Children whose families had difficulty obtaining services were six times more likely to have mental health problems than children not facing such difficulty. The finding was consistent with prior results (Bringewatt & Gershoff, 2010; Copeland & Snyder, 2011; Drabick et al., 2021; Santiago et al., 2013). When low-income families face challenges obtaining mental health care for children, as many often do, it may be chalked up to the unavailability of services in their area, or to their lack of transportation to available services, and/or to the need to pay for expensive services out-of-pocket (Cheng & Lo, 2025b; Koroloff et al., 1994, 1996; Overhage et al., 2024). Also, parents’ work schedules often pose barriers to accessing services for children during evening hours. In light of such challenges and barriers to needed mental health services, social workers and therapists can valuably help low-income families by finding free or low-cost programs and ensuring that their children (and troubled adult family members) become clients, and that they are familiar with practicable transportation options (or with in-home services, as appropriate) (Lakind & Atkins, 2018; Turner & Mueller, 2025).

Low-income children in the present study also had a relatively high risk for mental health problems if they had medical insurance through a public program. This result contradicted one prior study’s findings for a sample of Latinx children in San Francisco (Keeton et al., 2023). The present finding is plausibly explained by the fact that receiving needed mental health services via a public health insurance program helps secure diagnoses of mental health problems for low-income children.

## 5. Conclusions

This application of the multiple disadvantage model limitedly pinpointed that low-income children were more likely to have mental health problems when they experienced six crucial factors: families’ difficulty accessing needed mental health services, racial discrimination against Black children and Hispanic children residing in disadvantaged neighborhoods, children’s difficult relationships with parents and/or peers, children’s bullying victimization, children’s poor general health, and family members’ substance use. Low-income children’s risk of mental health problems was diminished when their parents enjoyed relatively robust mental health.

The application of a model to a nationally representative sample, as well as robust analysis, are strengths of the present study. It has four limitations as well, however. First, the NSCH researchers collected data from parents, not directly from children. Some children, though, may not discuss every experience or problem with their parents. Parents may, then, be misinformed—and they may also reflect personal bias when speaking of their children’s mental health. Second, there is a lack of standardized measures in this study. The measures employed here to quantify variables describing mental health problems, rundown neighborhoods, and unsafe neighborhoods were not standardized scales. The use of these proxy measures, while it helped secure the data to be analyzed, means that all efforts to generalize the present data analysis results must be undertaken cautiously. Third, the cross-sectional nature of this study’s data limits its implications surrounding causal relationships between the outcome and explanatory variables; indeed, such relationships might constitute reverse causality. Finally, some confounding factors, such as parental psychopathology, community support, and access to neighborhood resources, were not measured for the original dataset, which is a fourth limitation on the present analysis.

## Figures and Tables

**Table 1:** Descriptive statistics of low-income children (*n* = 7540).

	Percent	Mean	Range	Sd
Outcome variable				
Mental health problem (yes)	36.7			
(no)	63.3			
Social disorganization factors				
Rundown neighborhood (yes)	31.4			
(no)	68.6			
Safe neighborhood (1 = *definitely disagree*)		3.5	1–4	0.7
(2 = *somewhat disagree*)				
(3 = *somewhat agree*)				
(4 = *definitely agree*)				
Racial discrimination (yes)	6.0			
(no)	94.0			
Social structural factors				
White	51.4			
Black	11.6			
Hispanic	22.5			
Asian	5.8			
Other racial/ethnic minorities	8.7			
Girl	47.1			
Boy	52.9			
Child age: 6 to 10 years	38.7			
Child age: 11 to 13 years	24.7			
Child age: 14 to 17 years	36.6			
Parent age (years)		41.7	18–75	8.1
Parent education (1 = *8th grade or below*)		4.9	1–9	2.0
(2 = *9th–12th grade*)				
(3 = *graduated high school or GED*)				
(4 = *vocational school*)				
(5 = *some college*)				
(6 = *associate degree*)				
(7 = *undergraduate degree*)				
(8 = *master’s degree*)				
(9 = *doctoral or professional degree*)				
Employed parent (yes)	65.1			
(no)	34.9			
Family income-to-poverty ratio (%)		112.1	50–199	50.1
Social relationships				
Single mother (yes)	32.1			
(no)	67.9			
Family violence (yes)	8.0			
(no)	92.0			
Neighbor support (1 = *definitely disagree*)		9.4	3–12	2.2
(2 = *somewhat disagree*)				
(3 = *somewhat agree*)				
(4 = *definitely agree*)				
Poor parent-child communication (1 = *very well*)		1.5	1–4	0.7
(2 = *somewhat well*)				
(3 = *not very well*)				
(4 = *not well at all*)				
Child argued (1 = *never*)		2.1	1–4	0.9
(2 = *sometimes*)				
(3 = *usually*)				
(4 = *always*)				
Difficulty of parenting the child (1 = *never*)		5.3	3–15	2.2
(2 = *rarely*)				
(3 = *sometimes*)				
(4 = *usually*)				
(5 = *always*)				
Child’s difficulty with peers (1 = *no difficulty*)		1.3	1–3	0.6
(2 = *a little difficulty*)				
(3 = *a lot of difficulty*)				
Being bullied (yes)	34.5			
(no)	65.5			
Health/mental health				
Child general health (1 = *poor*)		4.4	1–5	0.8
(2 = *fair*)				
(3 = *good*)				
(4 = *very good*)				
(5 = *excellent*)				
Parent mental health (1 = *poor*)		3.8	1–5	1.0
(2 = *fair*)				
(3 = *good*)				
(4 = *very good*)				
(5 = *excellent*)				
Family mental health problem (yes)	13.4			
(no)	86.6			
Family substance use problem (yes)	13.0			
(no)	87.0			
Access to care				
Public health insurance	56.5			
Private health insurance	37.9			
Other health insurance	4.7			
Uninsured	7.7			
Difficulty of accessing mental health services (yes)	9.8			
(no)	90.2			

Note: sd = standard deviation.

**Table 2:** Logistic regression results on low-income children’s mental health problems (*n* = 7540).

Variables	OR	RSE	90% CI
Social disorganization factors			
Rundown neighborhood (no)	0.73 *	0.11	0.56–0.94
Safe neighborhood	0.78 *	0.09	0.65–0.93
Racial discrimination (no)	0.92	0.24	0.61–1.41
Social structural factors			
Black (White)	0.99	0.19	0.72–1.36
Hispanic (White)	0.72 *	0.13	0.53–0.97
Asian (White)	0.31 **	0.11	0.18–0.55
Other racial/ethnic minorities (White)	0.90	0.18	0.65–1.25
Girl (boy)	0.58 **	0.08	0.46–0.73
Child age: 6 to 10 years (age: 14 to 17 years)	0.90	0.13	0.71–1.15
Child age: 11 to 13 years (age: 14 to 17 years)	1.07	0.16	0.83–1.38
Parent age	0.99	0.01	0.98–1.01
Parent education	0.98	0.04	0.91–1.05
Employed parent (no)	0.91	0.12	0.73–1.12
Family income-to-poverty ratio	1.00	0.00	0.99–1.01
Social relationships			
Single mother (no)	1.24	0.18	0.98–1.57
Family violence (no)	1.11	0.22	0.80–1.54
Neighbor support	0.99	0.04	0.94–1.06
Poor parent-child communication	0.85	0.12	0.66–1.08
Child argued	1.17 *	0.09	1.03–1.33
Difficulty of parenting the child	1.13 **	0.05	1.05–1.21
Child’s difficulty with peers	3.29 **	0.35	2.77–3.91
Being bullied (no)	1.61 **	0.22	1.28–2.0
Health/mental health			
Child general health	0.74 **	0.06	0.64–0.86
Parent mental health	0.84 **	0.06	0.76–0.94
Family mental health problem (no)	1.21	0.22	0.90–1.63
Family substance use (no)	1.58 **	0.26	1.21–2.07
Access to care			
Public health insurance (uninsured)	1.78 **	0.30	1.35–2.35
Private health insurance (uninsured)	1.29	0.22	0.98–1.71
Other health insurance (uninsured)	1.58	0.47	0.97–2.59
Difficulty of accessing mental health services (no)	7.70 **	2.32	4.70–12.63
Wald’s χ^2^ =	562.52 **		

Notes: ** *p* < 0.01; * *p* < 0.05; OR = odds ratio; RSE = robust standard errors; CI = confidence interval; reference groups are in parentheses.

## Data Availability

The original data presented in the study are openly available in 2021 National Survey of Children’s Health (NSCH) dataset at https://www.census.gov/programs-surveys/nsch/data/datasets.html (accessed on 13 October 2025).
